# A new technique for treating hiatal hernia with gastroesophageal reflux disease: the laparoscopic total left-side surgical approach

**DOI:** 10.1186/s12893-021-01356-3

**Published:** 2021-10-09

**Authors:** Zhi Zheng, Xiaoye Liu, Chenglin Xin, Weitao Zhang, Yan Gao, Na Zeng, Mengyi Li, Jun Cai, Fandong Meng, Dong Liu, Jie Zhang, Jie Yin, Jun Zhang, Zhongtao Zhang

**Affiliations:** 1 Department of General Surgery, Beijing Friendship Hospital, Capital Medical University, 95 Yong-an Road, Xi- Cheng District, Beijing, 100050 China; 2 Beijing Key Laboratory of Cancer Invasion and Metastasis Research, Beijing, China; 3 National Clinical Research Center for Digestive Diseases, Beijing, China; 4 Beijing Institute of Clinical Medicine, Beijing, China; 5 Beijing Key Laboratory of Cancer Invasion and Metastasis Research, Department of Human Anatomy, School of Basic Medical Science, Capital Medical University, Beijing, China; 6 Clinical Epidemiology and Evidence-Based Medicine Unit, Beijing Friendship Hospital, Capital Medical University, Beijing, China; 7 Department of Gastroenterology, Beijing Friendship Hospital, Capital Medical University, Beijing, China; 8 Department of Ultrasonography, Beijing Friendship Hospital, Capital Medical University, Beijing, China; 9 Department of Radiology, Beijing Friendship Hospital, Capital Medical University, Beijing, China

**Keywords:** Total left-side surgical approach, Fundoplication, Hiatal hernia, Gastroesophageal reflux disease, Vagus nerve

## Abstract

**Introduction:**

Although the traditional bilateral surgical approach to treat hiatal hernia (HH) with gastroesophageal reflux disease (GERD) can provide local protection of the vagus nerve, the integrity of the entire vagus nerve cannot be evaluated. Therefore, we developed and described the total left-side surgical approach (TLSA), which theoretically reduces injury to the vagus nerve, and described the detailed surgical procedure.

**Methods:**

Initially, we performed a cadaver study to explore the characteristics of the vagus nerve. Then, we prospectively evaluated the TLSA in 5 patients with HH and GERD between June 2020 and September 2020. Demographic characteristics, surgical parameters, perioperative outcomes, and follow-up findings were analyzed.

**Results:**

The TLSA was successfully used in five patients (40–64 years old), and no major complications were noted. The median total operative time was 114 min, median blood loss was 50 mL, and median postoperative hospital stay was 3.8 days. Gastrointestinal function recovered within 4 days of surgery in all the patients. The 6-month follow-up gastroscopy examination showed well-established gastroesophageal flap valves. Compared with the baseline results, the 6-month follow-up results showed lower values for the total GerdQ score (12.4 vs. 6.2) and the total esophageal acid exposure time (3.48% vs. 0.38%). Based on the European Organization for Research and Treatment of Cancer quality of life questionnaire-stomach module 52 results, the incidence of dysphagia and flatulence decreased over time after the TLSA.

**Conclusions:**

The TLSA provides a clear and broad surgical field, less trauma, and rapid recovery; moreover, it is technically simple. Although our results suggest that the TLSA provides safety and short-term efficacy and is feasible for patients with HH and GERD, long-term results from a larger clinical trial are needed to validate these findings.

*Trial registration* ChiCTR2000034028, registration date is June 21, 2020. The study was registered prospectively

## Introduction

Hiatal hernia (HH) is a condition that involves herniation of abdominal contents into the mediastinum via the diaphragmatic hiatus and is characterized by a dilated esophageal hiatus [1]. HH is considered a major cause of gastroesophageal reflux disease (GERD), which may also be related to abnormal lower esophageal sphincter pressure [2]. Therefore, many patients with HH have GERD. Patients with GERD who do not respond to proton pump inhibitors (PPIs) may undergo laparoscopic HH repair with anti-reflux surgery [3, 4], which aims to reconstruct the local anatomical structures and address functional deficiencies at the esophagogastric junction [5]. The current primary treatment for HH with GERD involves the use of the traditional bilateral surgical approach (TBSA) to perform laparoscopic HH repair and Nissen fundoplication [6]. While this procedure can provide local protection of the vagus nerve, the integrity of the entire vagus nerve cannot be evaluated. Furthermore, the vagus nerve trunk and its branches are located in the lesser omentum, which must be incised during the TBSA. Although the vagus nerve trunk can be easily protected, small vagus nerve branches within the lesser omentum are usually not visible, thus leading to inadvertent injury during incision of the lesser omentum. These injuries can affect the patient’s reflux symptoms and quality of life [7, 8].

Therefore, we developed a new surgical approach that we called the “total left-side surgical approach” (TLSA) for laparoscopic HH repair with fundoplication. The potential advantages of the TLSA are that it is technically simple and preserves the lesser omentum and the hepatogastric ligament to a great extent based on the anatomical characteristics of the vagus nerve. This technique may also help improve the postoperative quality of life of patients with HH and GERD. This report describes our findings regarding the anatomical characteristics of the vagus nerve from a cadaver study, which we used to develop the TLSA. Further, we describe the detailed surgical procedure and discuss the preliminary results regarding the safety and short-term effectiveness of the TLSA in five patients who underwent surgical treatment for HH and GERD.

## Materials and methods

### Cadaver study

Chest and abdomen specimens were collected from deceased donors. Formalin-fixed cadaveric specimens were provided by Capital Medical University. This study was approved by the Ethics Committee of Beijing Friendship Hospital, Capital Medical University (2019-P2-060-02). The preliminary findings were used to evaluate the anatomical characteristics of the vagus nerve and the feasibility of the TLSA before it was used in patients.

### Registered clinical trial

We have initiated a prospective trial of laparoscopic TLSA for treating HH. The trial protocol is registered at the Chinese Clinical Trial Registry website (http://www.chictr.org.cn/index.aspx, ChiCTR2000034028) and was approved by the Ethics Committee of Beijing Friendship Hospital, Capital Medical University (L-2020-019). The clinical trial was launched in July 2020 and is scheduled to end in December 2023. During the research period, patients will be recruited from the Beijing Friendship Hospital for treatment, and they must provide written informed consent before undergoing surgery. We present the following article in accordance with the CONSORT reporting checklist.

### Inclusion criteria

The inclusion criteria are listed as follows which we described in previously work [9]. (1) Patients must have HH (type I/II/III) with GERD that is diagnosed via gastroscopy, high-resolution esophageal manometry, and 24-h esophageal pH monitoring. Patients must have undergone upper gastrointestinal radiography or abdominal computed tomography for assessment of disease severity. The study will also enroll the obesity patients. (2) Patients must have typical reflux symptoms, which do not respond well to standard medical therapy. (3) Patients must be 18–65 years old, regardless of their sex. (4) Patients must comply with the research protocol during the study period and provide informed consent. (5) Patients must be willing to attend follow-up visits and cooperate with the medical staff to collect relevant data.

### Exclusion criteria

Patients will be excluded from the study which we described in previously work [9]. (1) Patients with giant HH (type IV) (large hernia sacs are not suitable for the TLSA). (2) Patients who underwent cholecystectomy in the past. (3) Patients with severe uncontrolled recurrent infections or other severe uncontrolled concomitant diseases.

### Patients and follow-up

The present report covers the characteristics and short-term outcomes of five patients with HH and GERD who underwent surgical procedures via the TLSA at the General Surgery Department of Beijing Friendship Hospital affiliated to the Capital Medical University between June 2020 and September 2020. The patients were followed up for ≥ 6 months after the surgery via outpatient visits, inpatient visits, telephone contact, or mailed correspondence. The patient’s demographic characteristics, surgical parameters, perioperative outcomes, and follow-up findings were analyzed.

### Surgical methods

#### Surgeon positioning

**Fig. 1:**
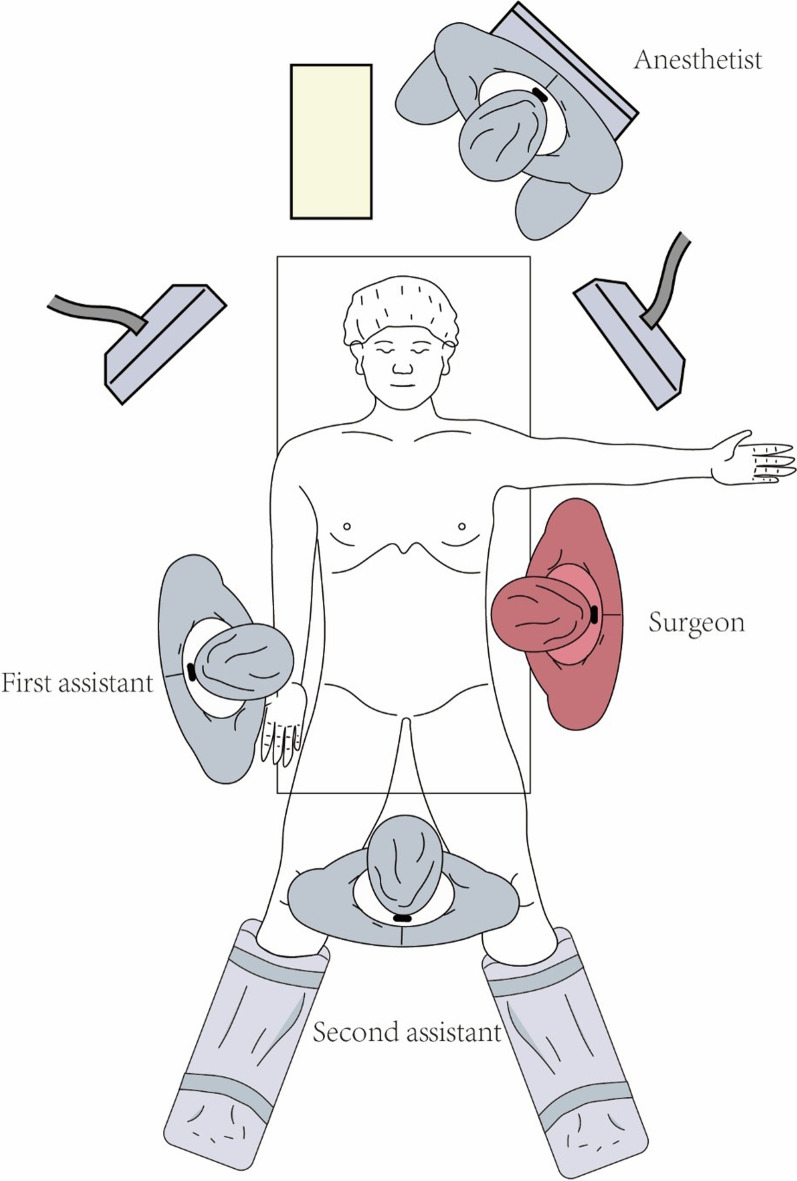
Surgeon position. The surgeon stands on the patient’s left side throughout the surgery, whereas the first assistant stands on the patient’s right side and the second assistant stands between the patient’s legs and manipulates the laparoscope. The picture depicted in Fig. 1 was my own based on the actual scenario of surgery

Based on the cadaver study’s results, we developed a new surgical approach for performing laparoscopic esophageal HH repair and fundoplication via the TLSA. The degree of fundoplication was determined based on the esophageal manometry and pH monitoring results. If the HH size was > 5 cm or if the diaphragm appeared weak on both sides of the defect, a surgical non-absorbable mesh was used. Surgery was performed with the patient in the supine position after successful general anesthesia induction and tracheal intubation. The surgeon stood on the patient’s left side throughout the surgery, and the first assistant stood on the patient’s right side. A second assistant stood between the patient’s legs to manipulate the laparoscope (Fig. 1).

#### Port placements

**Fig. 2:**
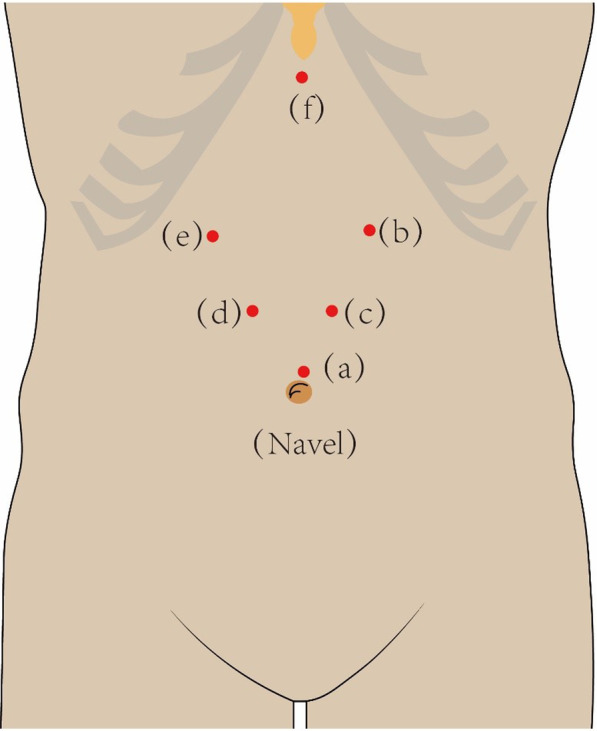
Puncture port placement. **a** A 12-mm trocar is inserted through a transverse incision, 1.0 cm above the umbilicus to establish the pneumoperitoneum and insert the laparoscope. **b** A left anterior axillary subcostal incision is created to accommodate the primary 12-mm trocar. **c** A left midclavicular horizontal incision is made 2.0 cm above the umbilicus to accommodate an auxiliary 5-mm trocar. **d** A right midclavicular horizontal incision is made 2.0 cm above the umbilicus to accommodate a 12-mm trocar for the assistant’s instruments. **e** A right anterior axillary subcostal incision is made to accommodate a 5-mm trocar. **f** An incision is made 2.0 cm below the xiphoid process to accommodate a 5-mm trocar that is used to expose the hiatal region. The picture depicted in Fig. 2 was my own based on the actual scenario of surgery

The port placements are detailed shown in Fig. 2 which we described in previously work [9].

#### Operative technique

**Fig. 3:**
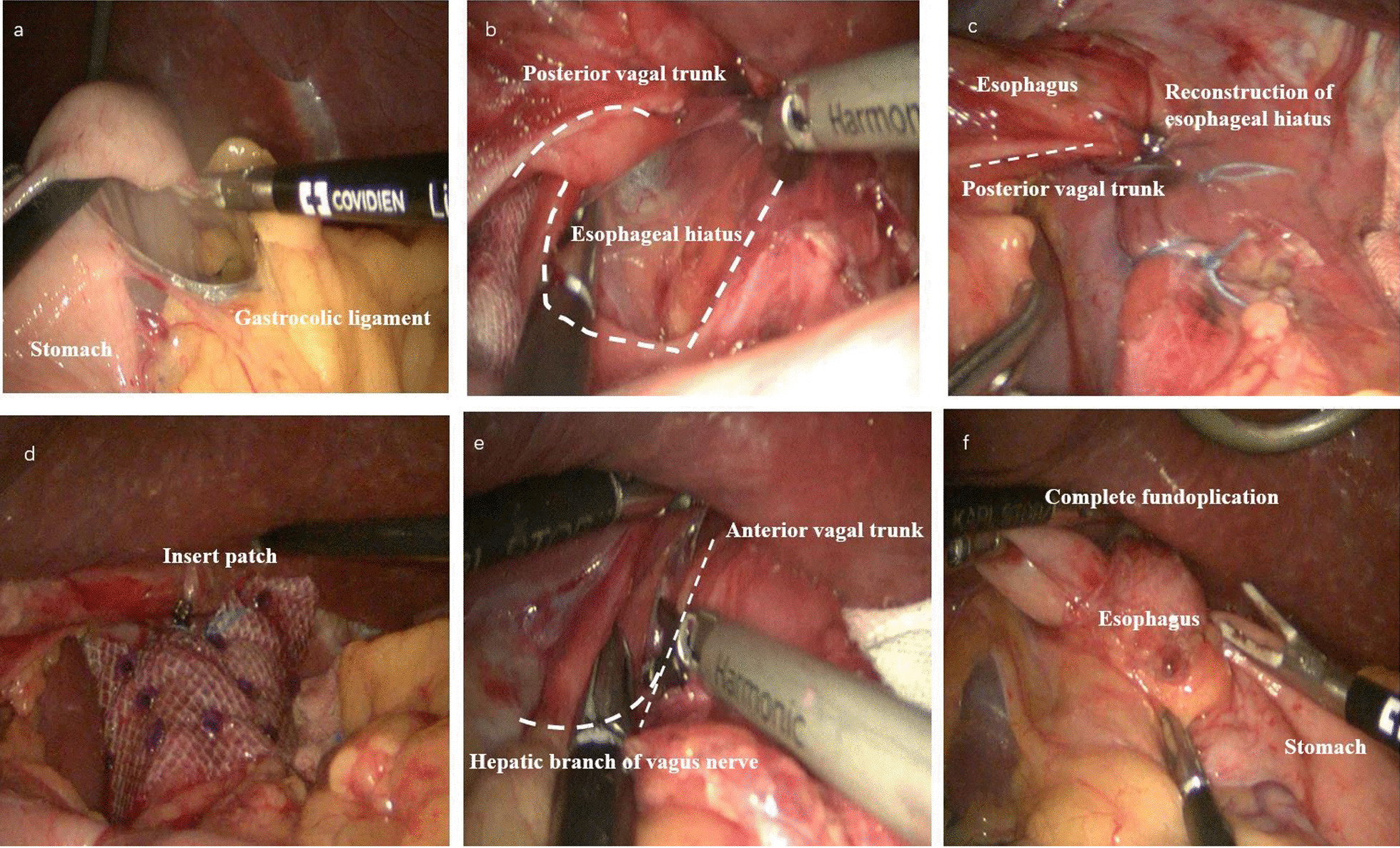
Operative technique. **a** On the stomach’s greater curvature, the gastrocolic ligament is incised along the avascular area between the left and right gastric omentum vessels, and the gastric fundus is lifted vertically toward the cardia to protect the vagus nerve. **b** The cardia, lower esophagus, and diaphragm are exposed, and the confluence of the left and right crus of the diaphragm is revealed. The retroperitoneum is incised at the left and right crus of the diaphragm, and the lower esophagus is dissociated for approximately 3–5 cm. The gastric fundus and the posterior wall of the esophagus are fully dissociated from the upper spleen. **c** Non-absorbable intermittent sutures are used at the left and right crus of the diaphragm to reconstruct the esophageal hiatus (diameter: approximately 1.5 cm). **d** The surgeon inserts the mesh and fixes it to the crus of the diaphragm with staples if the HH size is > 5 cm or the diaphragm on both sides of the defect is weak. **e** A small incision (approximately 2–3 cm) is made above the bifurcation of the anterior vagal trunk and the hepatic branch of the vagus nerve. This region is the avascular area of the lesser omentum. **f** The fundus of the stomach is rotated around the posterior aspect of the abdominal esophagus to the right anterior aspect of the esophagus (using non-absorbable sutures for 2 or 3 stitches intermittently) and then fixed to the right crus of the diaphragm and the right side of the esophagus. The left side of the gastric fundus is also sutured to the anterior esophagus and the left crus of the diaphragm, which avoids vagus nerve injury. Finally, the surgeon completes the fundoplication

The operative technique is shown in Fig. 3 which we described in previously work [9].

## Results

### Anatomical characteristics of the vagus nerve

The left and right branches of the vagus nerve are distributed in the anterior and posterior walls of the esophagus below the bronchial bifurcation. The left branch of the vagus nerve travels in the right anterior wall of the esophagus, where it forms the anterior trunk of the vagus nerve, and the right branch of the vagus nerve travels in the right posterior wall of the esophagus, where it forms the posterior trunk of the vagus nerve. Both branches pass through the esophageal hiatus of the diaphragm and give off additional nerve branches.

### Anterior vagal trunk

**Fig. 4:**
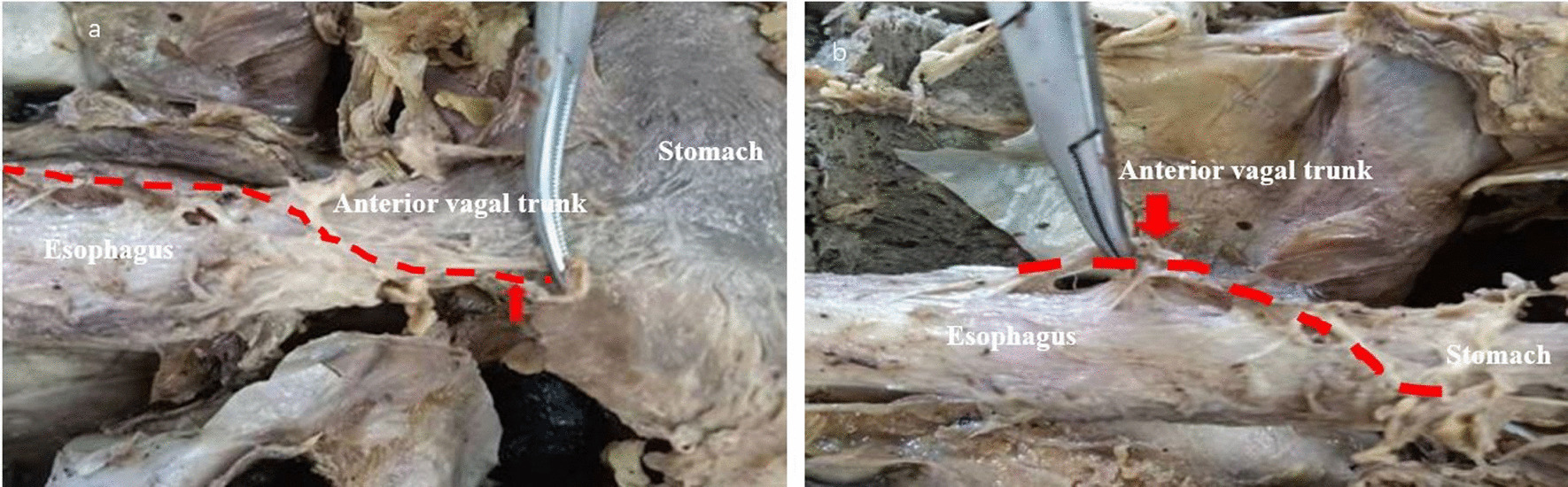
Anatomical characteristics of the anterior vagal trunk. **a** The anterior vagal trunk travels from the upper left to the lower right of the anterior esophageal wall (red dotted line). **b** It is located between the muscular layer and the peritoneum of the anterior abdominal esophageal wall, where it is closely adhered to the muscular layer of the esophagus (red dotted line)

The anterior vagal trunk is located between the muscular layer and the peritoneum of the anterior esophageal wall of the abdominal segment. A single trunk is a common trunk and travels from the upper left side to the lower right aspect of the anterior esophageal wall. It closely adheres to the muscular layer of the esophagus and can be easily damaged if it is not carefully identified during surgery. At the level of the cardia, the anterior vagal trunk divides into hepatic branches and into the anterior gastric branch. The hepatic branches mainly accompany the proper hepatic artery and travel to the hepatic portal system to join the hepatic plexus, which is distributed throughout the liver and the biliary tract, where it helps regulate the secretion activities of the hepatobiliary system. After dividing into the hepatic branch, the anterior vagal trunk continues to travel down the lesser curvature of the stomach to form the anterior gastric branch, which is often close to the lesser curvature of the stomach (< 1 cm). This branch travels down to the gastric angle and forms the anterior “crow’s claw” branch, which travels between the two peritoneal layers of the lesser omentum (Fig. 4).

### Posterior vagal trunks

**Fig. 5:**
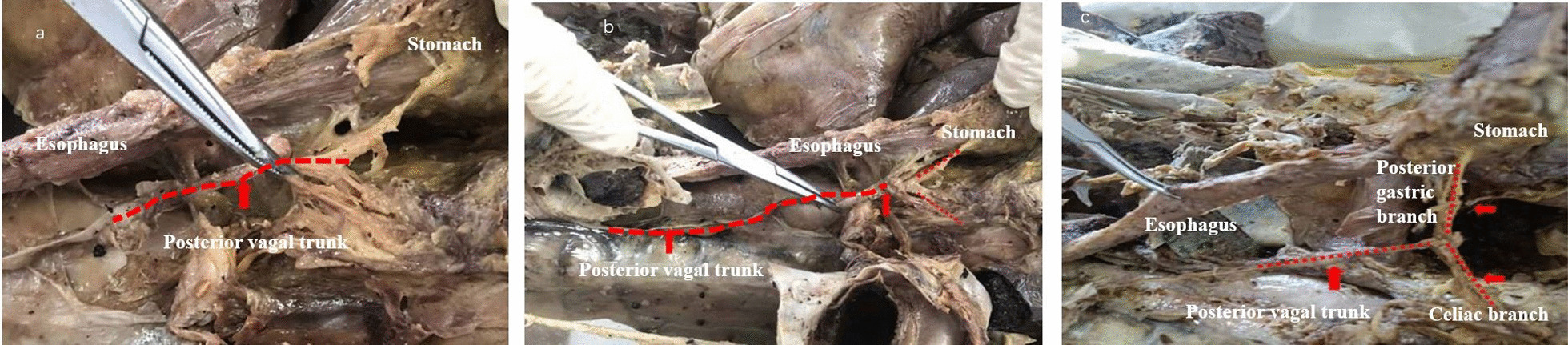
Anatomical characteristics of the posterior vagal trunk. **a** The posterior vagal trunk travels through the loose tissue outside the muscular layer of the right posterior wall of the abdominal esophagus (red dotted line). **b** The posterior vagal trunk produces nerve branches below the cardia (red dotted line) and **c** the celiac branch and posterior gastric branch (red dotted line)

The posterior vagal trunk is generally thicker than the anterior vagal trunk, and it travels through loose tissues outside the muscular layer of the right posterior wall of the abdominal esophagus. The posterior vagal trunk is simple to identify and dissect in the gastropancreatic fold, where it gives off the celiac branch below the cardia, which is closely related to the left gastric artery. The celiac branch travels diagonally to the lower right aspect of the abdominal ganglion, where it participates in the formation of the abdominal nerve plexus. After the celiac branch is removed, the posterior vagal trunk continues to travel inferiorly along the posterior wall of the stomach’s lesser curvature to form the posterior gastric branch, which adheres to the lesser curvature and continues to form the posterior “crow’s claw” branch at the gastric angle, where it further divides into 3 or 4 branches that are distributed to the posterior wall of the pylorus. The posterior vagal trunk and its branches are located in the triangular area created by the right crus of the diaphragm, the lateral margin of the stomach’s lesser curvature, and the left gastric artery (Fig. 5).

### Surgical outcomes

In our study, the demographic characteristics and operative results are summarized in Table 1. The procedures included Nissen fundoplication (1 patient), Toupet fundoplication (3 patients), and Dor fundoplication (1 patient). The mesh was inserted in all the patients for HH repair as the size of the defects was > 5 cm. The surgeon used meshes composed of a synthetic material, according to the hospital’s medical policy. The median total operative time was 114 min (range 60–150 min) and the median estimated blood loss was 50 mL; none of the patients required conversion to open surgery or a second surgery. Major complications, such as infection, bleeding, esophageal perforation, or death, were not reported. Gastrointestinal function, which was evaluated based on clinical symptoms, such as the first episode of exhaustion and need for oral feeding, recovered within 4 days of surgery in all the patients. The median postoperative hospital stay was 3.8 days (range 3–5 days).

**Table 1:** Patient demographic characteristics and operative results

Patient no.	Age (years)	BMI (kg/m^2^)	Fundoplication degree	Total operation time (min)	Mesh placement	Mesh material	Estimated blood loss (ml)	Time for gastrointestinal recovery (days)	Postoperative hospital stays (days)	Second operation
1	60–69	20.70	Toupet	150	Yes	Synthetic	20	2	3	No
2	50–59	30.86	Toupet	60	Yes	Synthetic	10	2	4	No
3	40–50	20.83	Nissen	120	Yes	Synthetic	10	4	3	No
4	60–69	26.04	Toupet	140	Yes	Synthetic	10	1	4	No
5	50–59	25.00	Dor	100	Yes	Synthetic	50	3	5	No

The patients attended a comprehensive follow-up visit after 6 months. Compared with the preoperative results, the follow-up endoscopic examination results showed that the Hill grade of the gastroesophageal flap valve had improved from grades 2–4 to grade 1 and that 2 patients experienced complete resolution of their esophagitis. Moreover, the mean total GerdQ score improved from 12.4 to 6.2 and the total esophageal acid exposure time improved from 3.48 to 0.38 %, which supports the short-term effectiveness of anti-reflux procedures performed with the TLSA (Table 2).

**Table 2:** Postoperative reexamination after 6 months

	Baseline				6-months follow-up			
Patient no.	Hill grade	LA	TEAE time (%)	Gerd-Q score	Hill grade	LA	TEAE time (%)	Gerd-Q score
1	I	–	1.4	10	I	–	0.8	6
2	I	–	5.7	14	I	–	0.3	6
3	II	–	1.2	12	I	–	0.3	6
4	IV	C	5.6	15	I	–	0.4	6
5	IV	B	3.5	11	I	–	0.1	7

*TATE time* total esophageal acid exposure time, *LA* Los Angeles classification

The incidence of complications was estimated using the European Organization for Research and Treatment of Cancer quality of life questionnaire-stomach module 52 at 1, 3, and 6 months after surgery (Table 3). Compared with the baseline results, dysphagia and flatulence scores had increased during the first month; however, these scores gradually decreased over time. There were no significant changes in abdominal pain.

**Table.3:** The STO52European Organization for Research and Treatment of Cancer quality of life questionnaire-stomach module 52 scores from baseline to the 6-month follow-up

	Baseline			1-month Follow-up			3-months Follow-up			6-months Follow-up		
Patient no.	Dysphagia	Flatulence	Abdominal pain	Dysphagia	Flatulence	Abdominal pain	Dysphagia	Flatulence	Abdominal pain	Dysphagia	Flatulence	Abdominal pain
1	1	2	2	1	2	2	1.3	2	1	1	1	1
2	1	1	1	1.3	2	1	1	3	2	1	2	1
3	1	2	2	2.7	2	2	2	3	2	1.3	1	2
4	1	1	1	2.3	3	2	1.3	1	1	1	1	1
8	1	1	3	1.3	3	2	1.3	1	2	1.3	1	1

### Gastroscopy findings

**Fig. 6:**
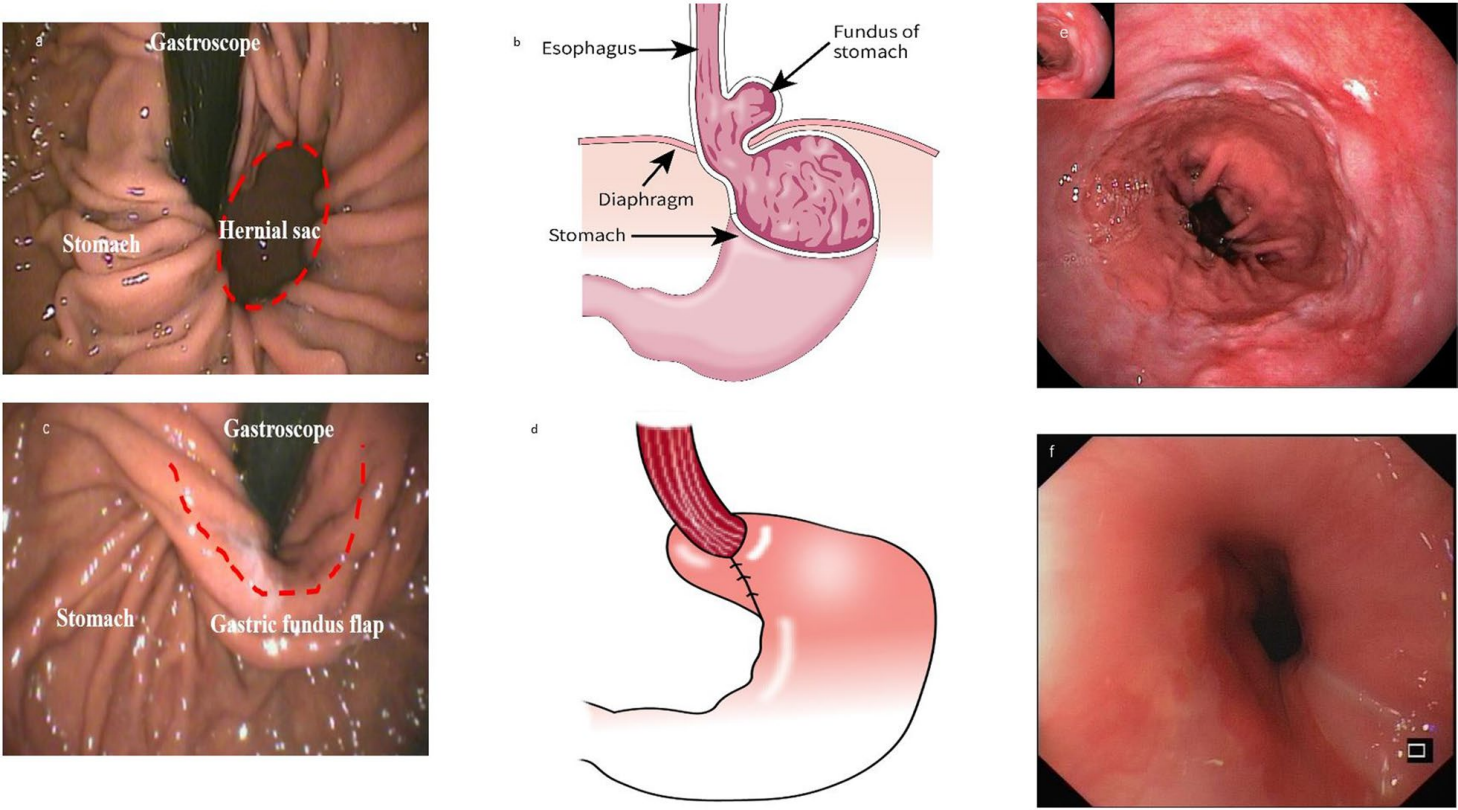
Comparing the findings from gastroscopy before surgery and 6 months after surgery. **a** Preoperative gastroscopy reveals a large hernia sac (red dotted circle) protruding into the chest. **b** A schematic diagram of the hiatal hernia. **c** The hernia sac disappeared after Nissen fundoplication, and the gastric fundus flap was visible (red dotted line). **d** A schematic diagram showing the results of the Nissen fundoplication. **e** Gastroscopy suggesting that the patient had severe esophagitis prior to the TLSA. **f** Gastroscopic examination 6 months after surgery indicating that the esophagitis has improved. The picture depicted in Fig. 6 was my own based on the actual gastroscopic photograph

All the 5 patients underwent gastroscopy before surgery and 6 months after surgery. Representative preoperative and postoperative gastroscopy findings from the one patient who underwent Nissen fundoplication are presented in Fig. 6. Preoperative evaluation revealed a large hernial sac protruding into the chest, which disappeared after the Nissen fundoplication. The gastric fundus flap was clearly visible at the 6-month follow-up. Furthermore, proper use of the TLSA resolved esophagitis in patients.

## Discussion

The Society of American Gastrointestinal and Endoscopic Surgeons issued guidelines for the management of HH in 2013 [10]; however, these guidelines did not provide detailed techniques for HH repair or discuss how to avoid nerve damage and protect organ functions during surgery. Moreover, cadaver studies and clinical trials on the treatment of functional diseases, such as HH and GERD, are still lacking [11, 12].

With the deepening of scholars’ understanding of HH, clinicians are paying more attention to improvements in postoperative quality of life of patients while pursuing curative effects, of which protection of the vagus nerve is particularly important. Studies have established that the hepatic branch of the vagus nerve regulates the movement of the liver and biliary tract [13, 14]. When the nerve is damaged, it leads to decreased gallbladder peristalsis, limited bile secretion, and gastrointestinal hormone secretion imbalance, which increases the incidence of gallstones and necrotizing cholecystitis [15, 16]. In severe cases, surgical intervention is required. Therefore, to reduce the incidence of gallstones, the hepatic branch of the vagus nerve needs to be effectively protected during surgery. In addition, the terminal branches of the vagus nerve control the peristaltic movements of the pylorus and duodenum, prevent the occurrence of gastric emptying disorders, reduce the secretion of gastric acid, and control gastric reflux to a certain extent [7, 17]. Therefore, when operating patients with functional disorders, injury to the vagus nerve is related not only to the expertise and skill of the surgeon but also to factors such as surgical approach, number of operations required, and anatomical variations of the vagus nerve. In our study, we have explained the anatomy of the vagus nerve in detail and have provided a reference for nerve protection in the treatment of functional disorders.

Advancements in laparoscopic tools and techniques and the associated advantages (less trauma, rapid recovery, and operative flexibility) have led to an increased use of minimally invasive techniques for treating HH. As a result, the primary treatment of HH now involves laparoscopic repair with fundoplication via the TBSA [18, 19]. The vagus nerve can be locally protected during procedures performed using the TBSA; however, the integrity of the entire vagus nerve cannot be evaluated. Thus, some cases involve unidentifiable injury to the hepatobiliary branch of the vagus nerve, which only becomes apparent after the patient experiences postoperative complications, such as bile secretion disorders and gastrointestinal dysfunction, that seriously affect their quality of life [5, 7, 14, 17].

Here, we performed a cadaver study to evaluate the anatomical characteristics of the vagus nerve and used the results to develop the TLSA, which is currently being tested in a clinical trial involving patients with HH and GERD. Through the present study, we found that the TLSA can theoretically preserve the physiological function of the vagus nerve and the organs it innervates, which is supported by the fact that none of our five patients experienced any postoperative adverse effects or complications. Moreover, compared with the TBSA, the TLSA permits full dissociation of the gastrosplenic ligament at the stomach’s greater curvature, which allows the surgeon to stretch the stomach to the right and, thus, obtain a broader surgical field. The increased working area may permit better outcomes in terms of laparoscopic hiatus reconstruction, mesh placement, suturing, and fixation, especially in bariatric and morbidly obese patients. Finally, the TLSA may also help shorten the operation time, reduce surgical trauma, and improve short-term therapeutic effects and postoperative quality of life.

Both TBSA and TLSA can be used in function-preserving procedures, however the TLSA is a modification of the TBSA. The significant difference between the two is that the TLSA can reduce the involvement of the lesser omentum, so that the vagus trunk and its branches that are distributed in the lesser omentum can be protected to the maximum extent possible, thereby decreasing the probability of injury to the vagus nerve.

At present, neurophysiological monitors are widely used in the management of thyroid and orthopedic diseases with good efficacy; however, their use in the treatment of HH is still controversial. In their study involving mice, Berthoud et al. used neurophysiological monitors to discover that the hepatic branch of the vagus nerve is mainly distributed in the distal stomach and the celiac branch is distributed in the duodenum [20]. Based on the anatomical structure of the vagus nerve in mice, a clinical trial conducted by Korean scholars found that the nerve endings of the hepatic and celiac branches are mainly distributed in the duodenum [21]. During surgery, electrical stimulation acts on the hepatic and celiac branches of the vagus nerve. By recording the surface electrical activity of the duodenum, the integrity of the vagus nerve can be effectively determined, which suggests that electrophysiological evaluation of nerves has some practical implication in the protection of the vagus nerve around the stomach[21]. Although we cannot directly evaluate the integrity of the vagus nerve after surgery, we can indirectly evaluate its function based on the improvement in patient’s postoperative clinical symptoms, quality of life, and long-term gallbladder stone incidence, which can confirm the viability of the TLSA. In the future, the neurological function of the vagus nerve can be preserved to a greater extent with the help of neurophysiological monitors.

This study has several strengths. First, it is a prospective study; therefore, the efficacy and safety endpoints to be studied were clearly and completely recorded, guaranteeing high quality and objectivity of the data. Second, the successful treatment and progress of patients in this study will encourage treatment of patients with HH and GERD using a novel approach. Lastly, the TLSA procedure was developed based on the results of the cadaver study, which has laid a theoretical foundation for the implementation of the TLSA.

This study has some limitation. Our findings are limited due to its single-center design, small sample size, and a short follow-up period. In addition, this is an observational study. Although the safety and efficacy of the TLSA can be demonstrated to some extent, the study lacks a comparison with the clinical data of procedures performed using the TBSA. Therefore, a multi-centered prospective trial which we conducted currently that includes long-term follow-up periods and a large sample to compare the outcomes of the TLSA and TBSA to validate our preliminary findings of good safety and efficacy [9].

## Conclusions

The TLSA provides a broad and clear surgical field, less trauma, and rapid recovery; moreover, it is a technically simple surgery, which may make it especially suitable for obese patients, and patients with substantial comorbidities. The findings of this study will allow for a safer and simpler treatment of patients with HH and GERD, without compromising the structure and function of the vagus nerve to a certain extent.

## Data Availability

Data sets are available by request to the corresponding author.

## Abbreviations

TLSA: Total left-side surgical approach
HH: Hiatal hernia
GERD: Gastroesophageal reflux disease
PPIs: Proton pump inhibitors
TBSA: Traditional bilateral surgical approach
